# Impact of antidepressant use on survival outcomes in glioma patients: A systematic review and meta-analysis

**DOI:** 10.1093/noajnl/vdae181

**Published:** 2024-10-26

**Authors:** Yulu Ge, Yaning Cao, Qi Wang, Yu Wang, Wenbin Ma

**Affiliations:** Eight-year Medical Doctor Program, Chinese Academy of Medical Sciences and Peking Union Medical College, Beijing, China; Department of Neurosurgery, Center for Malignant Brain Tumors, National Glioma MDT Alliance, Peking Union Medical College Hospital, Chinese Academy of Medical Sciences and Peking Union Medical College, Beijing, China; Eight-year Medical Doctor Program, Chinese Academy of Medical Sciences and Peking Union Medical College, Beijing, China; Department of Neurosurgery, Center for Malignant Brain Tumors, National Glioma MDT Alliance, Peking Union Medical College Hospital, Chinese Academy of Medical Sciences and Peking Union Medical College, Beijing, China; Eight-year Medical Doctor Program, Chinese Academy of Medical Sciences and Peking Union Medical College, Beijing, China; Department of Neurosurgery, Center for Malignant Brain Tumors, National Glioma MDT Alliance, Peking Union Medical College Hospital, Chinese Academy of Medical Sciences and Peking Union Medical College, Beijing, China; Department of Neurosurgery, Center for Malignant Brain Tumors, National Glioma MDT Alliance, Peking Union Medical College Hospital, Chinese Academy of Medical Sciences and Peking Union Medical College, Beijing, China

**Keywords:** antidepressants, depression, glioma, overall survival

## Abstract

**Background:**

Depression is common among glioma patients, and antidepressants are frequently prescribed to manage symptoms. Understanding the impact of antidepressants on glioma patient survival is crucial for informing treatment strategies.

**Methods:**

A systematic search was conducted in PubMed and EMBASE databases for studies published from January 1994 to March 2024. The search strategy included terms related to overall survival, prognosis, antidepressants, and gliomas. A manual search was performed in the reference lists. According to the preferred reporting items for systematic reviews and meta-analyses (PRISMA) guideline, 2 authors independently extracted data. Statistical analysis was performed using Review Manager (version 5.4.1) software, employing a random effects model based on study heterogeneity. The primary outcome was overall survival (OS). Hazard ratios (HRs) were used to present survival differences between the 2 arms. HRs after correcting for confounders were prioritized for extraction.

**Results:**

Seven retrospective cohort studies involving 5579 patients were analyzed. Selective serotonin reuptake inhibitors (SSRIs) showed no significant survival difference in all glioma patients (HR = 1.34, 95% confidence interval [CI]: 0.66–2.70) and in GBM patients (HR = 1.05, 95% CI: 0.45–2.46), while non-SSRIs had an unfavorable impact on OS in GBMs (HR = 3.54, 95% CI: 2.51–4.99). When considering LGG, both SSRIs and non-SSRIs usage demonstrated associations with poorer survival outcomes (SSRIs: HR = 3.26, 95%CI: 2.19–4.85; Non-SSRIs: HR = 7.71, 95% CI: 4.25–14.00).

**Conclusions:**

Antidepressant use was not significantly associated with better survival outcomes, emphasizing the need for reconsidering the real effects of antidepressant medication. Future clinical research should address patient heterogeneity to better clarify the effects of antidepressants on glioma survival.

**Key Points**

- Antidepressants are not significantly associated with improved survival outcomes in gliomas.
- Future clinical research should more carefully consider patient heterogeneity.

**Importance of the Study**

Depression is common among glioma patients, and antidepressants are frequently prescribed to manage symptoms. However, prior clinical research has shown conflicting results regarding the impact of antidepressants on glioma prognosis, with some studies indicating potential benefits while others suggest adverse effects. Our comprehensive meta-analysis reveals that selective serotonin reuptake inhibitors (SSRIs) use showed no significant survival difference, while non-SSRIs were associated with an unfavorable overall survival in glioblastomas. When considering low-grade gliomas, both SSRIs and non-SSRIs usage demonstrated associations with poorer survival outcomes. Therefore, antidepressant use seems not significantly associated with better survival outcomes, emphasizing the need for reconsidering the real effects of antidepressant medication. Future clinical research should address patient heterogeneity, including matching patients’ depressive states, recording antidepressant dosages along with concurrent treatment regimens, and distinguishing between different types of antidepressants, to better clarify the effects of antidepressants on glioma survival.

Gliomas, the most common primary malignant brain tumors, have an annual incidence rate of 6.5 per 100 000 individuals.^1^ At the time of presentation, patients typically exhibit focal symptoms such as hemiparesis, hemisensory loss, and visual field deficits, and alternatively exhibit less localized symptoms such as cognitive impairment.^2^ The standard treatment regimen for gliomas involves maximal surgical resection combined with adjuvant radiotherapy and chemotherapy.^3^ However, despite these interventions, patient survival remains poor. The most aggressive subtype, glioblastoma multiforme (GBM), is associated with a median survival of only 14.6 months.^3^ The clinical symptoms, along with the resultant decline in quality of life and dismal prognosis, not only inflict physical suffering upon patients but also elicit significant emotional distress. Glioma patients are significantly more likely to be depressed than the general population, having a median point prevalence of 16%–41% for depression during tumor treatment as assessed by self-report questionnaires.^4^ Due to the high prevalence of depression, treatment with antidepressants is more prevalent among glioma patients, with approximately 27% of individuals receiving such therapy.^5,6^

Psychotropic drugs can penetrate the blood-brain barrier and regulate the levels of neurotransmitters and neuromodulators, thus impacting neuronal activity and altering mental states. Interestingly, despite the initial intent to alleviate depression, antidepressants were found to be capable of inhibiting the malignant behavior of glioma cells. As early as 2005, it was found that paroxetine and fluoxetine, selective serotonin reuptake inhibitors (SSRIs), and clomipramine, a TCA, could cause apoptosis in rat glioma cell line.^7^ Subsequent research delved deeper into investigating the real role that certain antidepressants could play. Some researchers found that fluoxetine could suppress EGFR signaling, a feature that other kinds of SSRIs do not present.^8^ In the GBM39 mouse model, fluoxetine at human-equivalent doses of 50 and 80 mg/day significantly inhibited tumor growth and prolonged survival in a dose-dependent manner, whereas a low dose equivalent to 20 mg/day did not affect tumor growth. Fluoxetine was administered to mice once daily via oral gavage from the time the tumors were established until the animals’ death. Additionally, fluoxetine at around 50 mg/day significantly enhanced the survival benefits of TMZ therapy in mouse models.^8^ It is important to note, however, that most patients with depression are treated with a low dose of 20 mg/day. Similarly, imipramine, a TCA, inhibits ERK/NF-κB signaling, thereby impeding glioma cell invasion, angiogenesis, and proliferation, and this therapeutic efficacy was validated in a glioblastoma-bearing mouse model (10 mg/kg imipramine every day for mouse).^9^ In summary, numerous studies have elucidated the anti-glioma effects of some kinds of antidepressants at the preclinical level.^10–15^

However, despite the promising findings from preclinical studies, conclusions regarding whether antidepressants can prolong patient survival in clinical cohort studies are inconsistent. In all the articles we retrieved, a total of 7 retrospective cohort studies were found to investigate the relationship between antidepressant use and patient survival. The study population included were summarized in Table 1. Among them, one have reported a beneficial effect of antidepressant use on patient prognosis,^8^ while others have reported no association^16–19^ or have been associated with reduced survival.^5,20^ In particular, a recent large retrospective study of 1231 individuals reported that patients taking antidepressants had significantly worse survival.^5^ Positive conclusions drawn from preclinical studies of antidepressants may offer a potential improvement in patient prognosis. However, the interplay of various factors complicates this potential benefit. For instance, the depressive state itself negatively impacts patient survival,^21^ and the associated reduction in quality of life due to depression further exacerbates this issue.^22^ These counteracting factors likely contribute to the mixed outcomes observed in existing cohort studies.

**Table 1.** An Overview of the 7 Included Studies

Study	Time frame	Country	Data sources	Types of antidepressants evaluated	Sample size	
					All patients	With antidepressants use
Bi J, et al. 2021.	2003–2017	US	the IBM MarketScan insurance claims dataset	Fluoxetine	192 GBMs	10 (5.2%)
Caudill JS, et al. 2011.	1999–2008	US	Institutional records^a^	SSRI	160 GBMs	35 (21.9%)
Edström S, et al. 2023.	2009–2013	Sweden	The RISK North database	SSRI, non-SSRI	754 GBMs 477 LGGs	GBM: 205 (27.2%) LGG: 141 (29.6%)
Gramatzki D, et al. 2020.	2005–2014	Switzerland	The Cancer Registry of the Cantons Zurich and Zug	Antidepressant drug	404 GBMs	65 (16.1%)
Otto-Meyer S, et al. 2020.	2000/1/1–2018/3/8	US	The Northwestern Medicine Enterprise Data Warehouse (EDW)	SSRI	497 GBMs	151 (30.4%)
Seliger C, et al. 2023.			The CENTRIC, CORE, AVAglio and ACT-IV trials	Antidepressant drug	1731 GBMs	146 (8.4%)
Walker AJ, et al. 2012.	1987–2010	UK	The General Practice Research Database	Tricyclics	1364 Gliomas	57 (4.2%)

Hence, it is crucial to consolidate and comprehensively analyze existing research findings. Our current meta-analysis endeavors to determine whether glioma patients indeed derive survival benefit from antidepressant treatment, elucidate which specific glioma subgroups may benefit, and identify which particular class of antidepressants confers benefits. This endeavor aims to provide valuable insights to better inform the clinical management of depressive symptoms in glioma patients. In addition, after systematically searching and reviewing the literature, we provided a more comprehensive understanding of the existing antidepressant drug studies and tried to identify some potential deficiencies, hoping to offer better guidance for future studies.

## Materials and Methods

### Search Strategy and Study Eligibility

According to preferred reporting items for systematic reviews and meta-analyses (PRISMA) criteria, an online systemic search was conducted through online databases (PubMed and EMBASE). The search strategy was “([overall survival] OR [prognosis]) AND ([Antidepressive Agents] AND ([glioma] OR [glioblastoma]).” The time frame of the search was from January 1994 until March 2024. In addition, a manual search was performed in the reference lists of the selected papers and the excluded review articles to avoid missing any eligible publications. When it was necessary to contact the authors or other experts, the corresponding author’s e-mail address was retrieved from the article and an e-mail contact was sent.

Two authors blindly participated in the literature review. In cases of disagreement between them, a third author was involved in making the final decision. The inclusion criteria were: (1) English publications; (2) controlled studies, either randomized or non-randomized, with one arm for glioma patients with antidepressant use and the other arm for glioma patients without antidepressant use; (3) survival data reported. The exclusion criteria were: (1) reviews; (2) case reports; (3) letters to editors and editorial comments; (4) conference abstract; (5) repeated publications from the same author or from the same center; (6) non-English articles. All initial results underwent staged selection and screening, the first stage by assessing the title and abstract to exclude unrelated articles, reviews or meta-analyses, editorial comments, and case reports. The second stage was conducted by full-text assessment to exclude repeated publications and non-controlled case series.

### Quality Appraisal and Risk of Bias Assessment

Quality appraisal was initially conducted by 3 authors (Y.G., Y.C., and Q.W.) independently using the CASP Qualitative Research Checklist tool as a screening tool. The CASP Checklist for a cohort study^23^ allowed each paper to be appraised by the researchers to determine the validity of the results, including assessing the risk of bias. Each included paper was assessed systematically using the CASP Checklist. Following this, a joint meeting of authors was held to discuss and come to an agreement on the quality of each paper. Also, the Newcastle-Ottawa Scale (NOS) was employed in this meta-analysis to assess the quality of non-randomized trials. Scores of 7–9, 4–6, and 4 were classified as having a low, moderate, or high risk of bias, respectively. Low-quality studies would be excluded. Scoring was done separately and blindly by 2 researchers (Y.G. and Y.C.) and a third one (Q.W.) was involved in deciding when there was disagreement.

### Data Extraction

Based on sound clinical principles, the following variables were independently and blindly extracted by 2 authors (Y.G. and Y.C.) and checked by a third one (Q.W.): total number of patients, number of patients with antidepressants use, types of antidepressant drugs, strategies for assessing antidepressants use, median overall survival in days, hazard ratio and its 95% confidence interval. Any discrepancy in the extracted data were dissolved after discussion among all the authors.

### Outcomes

The primary outcome of this systematic review and meta-analysis was to compare overall survival between glioma patients with or without antidepressant use. Hazard ratios (HRs) were used to present survival differences between the 2 arms. Given that confounders, such as age, Karnofsky Performance Status, and extent of resection, can bias results, we prioritized extracting HRs of antidepressants after correcting for confounders in the selected articles. The specific adjusted confounders of each article were summarized in Supplementary Table 2.

### Statistical Analysis

Review Manager (RevMan) software (version 5.4.1) was employed for statistical analysis and the creation of forest plots for this meta-analysis. The mean difference with the 95% confidence interval (CI) was utilized for continuous data (Hazard Ratio). The I^2^ value was used to determine the heterogeneity of the research. For I^2^ less than 50%, the fixed effect model was utilized. For I^2^ more than 50%, the random effect model was utilized to correct the weights in the fixed effect model, in order to minimize the effect of heterogeneity among the studies. The Z-test was used to assess the overall impact. *P*-values less than .05 were deemed significant in all tests.

## Results

### Search Results

**Figure 1.**
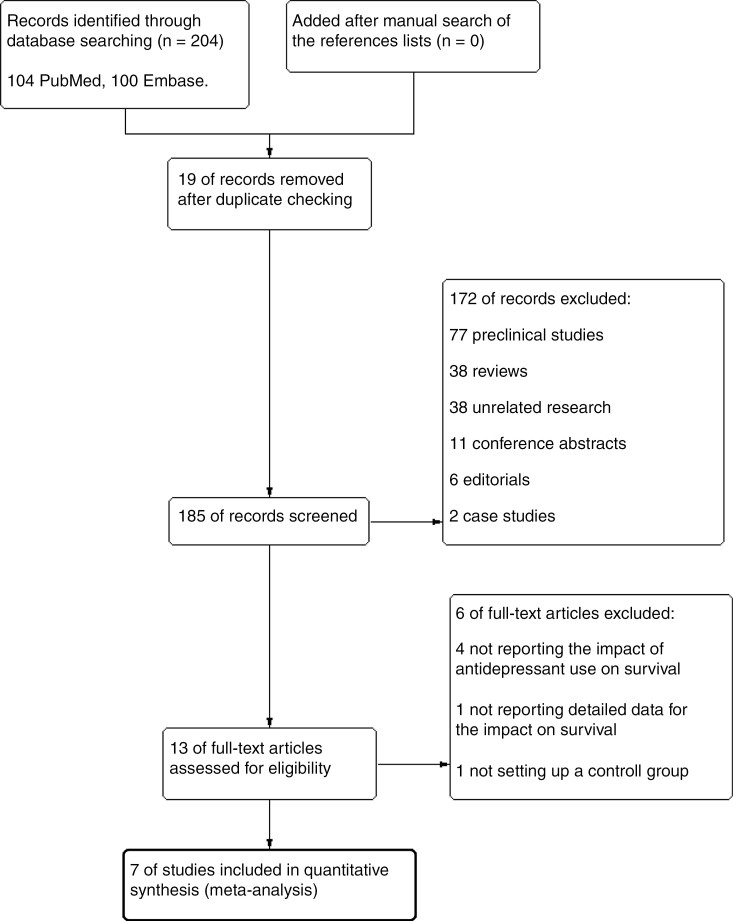
The flow diagram demonstrates the screening and selection processes.

The flow of the screening and selection processes is demonstrated in the PRISMA diagram in Figure 1. The initial search in the electronic databases displayed 204 articles, which underwent initial assessment, and 191 of which were excluded. A further 6 records were excluded in the second stage of the full-text assessment. Four of these did not report an effect of antidepressants on survival.^24–27^ One, while reporting that antidepressant use did not significantly affect survival, did not provide specific survival data.^28^ One did not have a control group and only provided survival times for the single arm of the group taking the medication.^29^ Eventually, 7 non-randomized, observational, retrospective cohort studies with 5579 glioma patients were included.

Table 1 provides an overview of the included studies. Table 2 provides an overview of the baseline characteristics and treatment regimens of the patient populations included in each analysis. The specific strategies of these studies for assessing antidepressants use were summarized in Supplementary Table 1. Of the total of 5579 patients, 810 (14.5%) had antidepressive medications. Two studies grouped all antidepressants together and analyzed their effects on survival.^18,20^ Four studies analyzed the effects of SSRIs or non-SSRIs,^5,16,17^ and one of them specifically analyzed 3 types of SSRIs, ie, fluoxetine, citalopram, and escitalopram.^30^ One study discussed tricyclic drugs.^19^

**Table 2.** Baseline Characteristics Of Patient Population Included in Each Study

	Patients with antidepressants use									
Study	Sample size	Mean age	Gender		Operation			First-line therapy		
			Male	Female	Biopsy	Incomplete resection	Gross total resection	RT plus TMZ	RT alone	Chemotherapy alone
Bi J, et al. 2021.	10							10 (100%)		
Caudill JS, et al. 2011.	35	55.5	18 (51.4%)	17 (48.6%)	14 (40.0%)	12 (34.3%)	9 (25.7%)			
Gramatzki D, et al. 2020.	65	61.3	40 (61.5%)	25 (38.5%)	14 (21.5%)	45 (69.2%)	6 (9.2%)	33 (50.8%)	11 (16.9%)	2 (3.1%)
Otto-Meyer S, et al. 2020.	151	59.7	83 (55.0%)	68 (45.0%)	15 (9.9%)	136 (90.1%)		TMZ used in all patients RT not known		
Seliger C, et al. 2023.	146		71 (48.6%)	75 (51.4%)	82 (56.2%)		64 (43.8%)	146 (100%)		
Walker AJ, et al. 2012.	57	56.7	18 (31.6%)	39 (68.4%)						
	**Patients without antidepressants use**									
Study	Sample Size	Mean Age	Gender		Operation			First-line therapy		
			Male	Female	Biopsy	Incomplete Resection	Gross total resection	RT plus TMZ	RT alone	Chemotherapy alone
Bi J, et al. 2021.	182							182 (100%)		
Caudill JS, et al. 2011.	125	57.0	86 (68.8%)	39 (31.2%)	34 (27.2%)	58 (46.4%)	33 (26.4%)			
Gramatzki D, et al. 2020.	339	62.8	216 (63.7%)	123 (36.2%)	79 (23.4%)	202 (59.8%)	56 (16.6%)	170 (50.1%)	65 (19.2%)	25 (7.4%)
Otto-Meyer S, et al. 2020.	346	59.1	216 (62.4%)	130 (37.6%)	71 (20.5%)	275 (79.5%)		TMZ used in all patients RT not known		
Seliger C, et al. 2023.	1585		955 (60.3%)	630 (39.7%)	857 (54.1%)		725 (45.7%)	1585 (100%)		
Walker AJ, et al. 2012.	1307	45.5	760 (58.2%)	547 (41.8%)						
	**All patients**									
Study	Sample Size	Mean Age	Gender		Operation			First-line therapy		
			Male	Female	Biopsy	Incomplete resection	Gross total resection	RT plus TMZ	RT alone	Chemotherapy alone
Edström S, et al. 2023.	LGG	477	59	267 (56%)	210 (44%)	124 (26%)	353 (74%)			
	GBM	754	64	460 (61%)	294 (39%)	204 (27%)	550 (73%)			

All available information about the patient’s baseline characteristics and treatment regimen is listed in this table. RT, radiotherapy; TMZ, temozolomide; LGG, low-grade glioma; GBM, glioblastoma.

Quality appraisal using the CASP Checklist tool revealed that all selected studies were of medium or high quality. In short, these studies addressed a clearly focused issue and the cohorts were recruited in an acceptable way. The exposure and outcome could be accurately measured to minimize bias. Each article corrected for confounders when analyzing the impact of antidepressants on survival. Moreover, A NOS risk of bias evaluation revealed that all studies are within the low-risk category (Table 3). Caudill JS, et al.’s study lost 0.5 score for relatively small and regionalized samples. All the included studies failed to gain one score for adequacy of follow-up, owing to the non-reporting of the number of lost visitors and the lost reasons, except for Seliger C, et al.’s study which collected data from 4 registered clinical trials. Overall, all these 7 studies were classified as having a low risk of bias.

**Table 3.** Quality Assessment of Included Studies Using Newcastle-Ottawa Scale

Study	Selection				Comparability	outcome			Overall
	Representativeness of the exposed cohort	Selection of the non-exposed cohort	Ascertainment of exposure	Outcome of interest not present at start		Assessment of outcome	Adequate follow-up length	Adequacy of Follow-Up	
Bi J, et al. 2021.	1	1	1	1	2	1	1	0	8
Caudill JS, et al. 2011.	0.5	1	1	1	2	1	1	0	7.5
Edström S, et al. 2023.	1	1	1	1	2	1	1	0	8
Gramatzki D, et al. 2020.	1	1	1	1	2	1	1	0	8
Otto-Meyer S, et al. 2020.	1	1	1	1	2	1	1	0	8
Seliger C, et al. 2023.	1	1	1	1	2	1	1	1	9
Walker AJ, et al. 2012.	1	1	1	1	2	1	1	0	8

### Overall Survival

**Figure 2.**
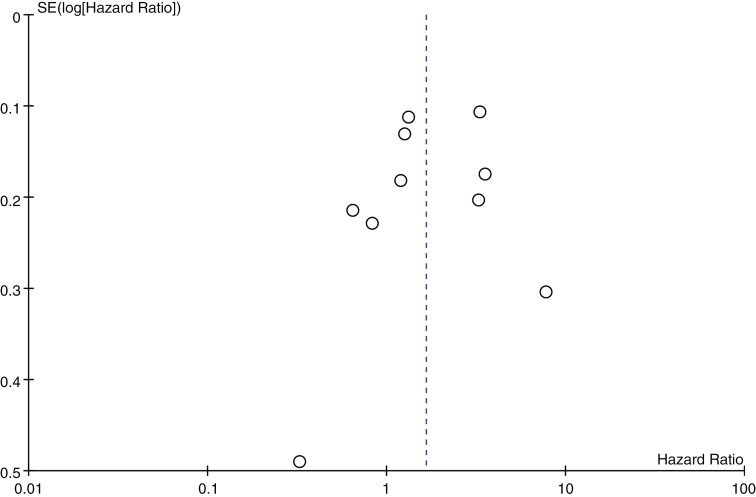
The funnel plot of all the survival data was obtained. Of the 7 articles included, the study by Edström S, et al. was divided into 4 distinct sections, each considered separately in the analysis. Consequently, this study contributed 4 data points, resulting in a total of 10 data points in the funnel plot.

Data from Edström S, et al.’s study was divided into 4 sections for inclusion in subsequent survival analyses (GBM, SSRI; GBM, non-SSRI; Grade 2–3, SSRI; Grade 2–3, non-SSRI), given that this research provided survival analyses for each of these 4 sections separately, and overall data were not available. Figure 2 illustrates a funnel plot consisting of all the survival data obtained, where the data points are roughly symmetrical. Therefore, publication bias could be considered relatively low.

#### The impact of SSRIs on the overall survival of glioma patients.—

**Figure 3.**
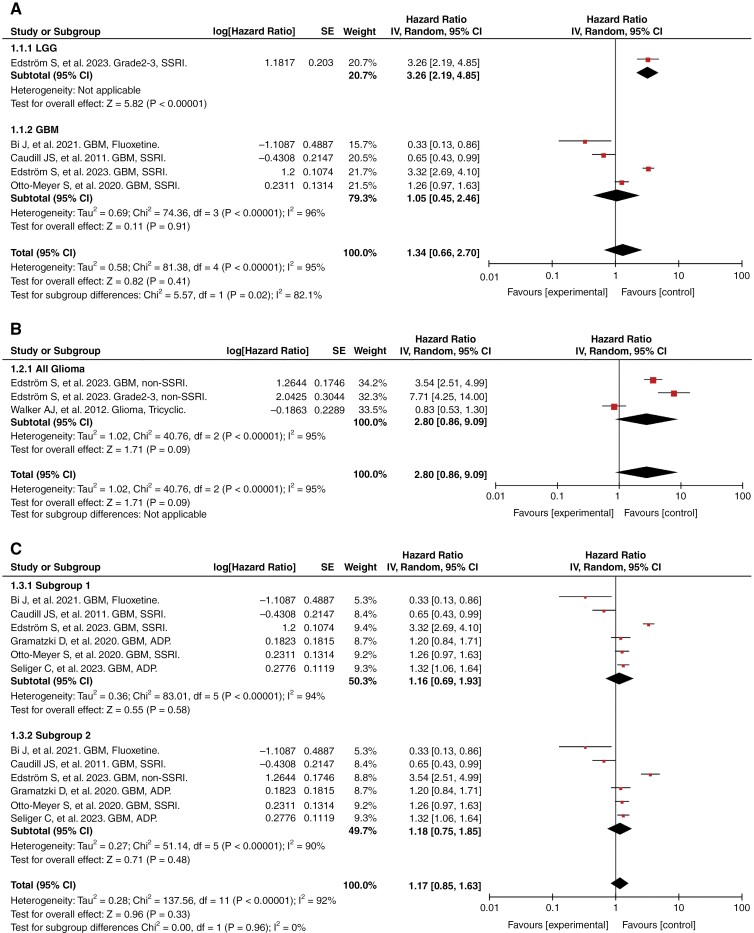
The impact of antidepressants on overall survival of glioma patients. (A) The impact of SSRIs on overall survival of GBM patients and LGG patients. (B) The impact of non-SSRIs on overall survival of glioma patients. (C) The impact of overall antidepressants on overall survival of GBM patients. (SSRI, selective serotonin reuptake inhibitors; GBM, glioblastoma; LGG, low-grade glioma)

Four studies involving 1603 GBMs and 477 LGGs compared OS between glioma patients with or without SSRIs use. Overall, when considering all grades of gliomas, there was no significant survival difference (Figure 3A, HR = 1.34, 95% CI: 0.66–2.70). A similar result was obtained when only considering GBM (Figure 3A, HR = 1.05, 95% CI: 0.45–2.46). However, SSRIs seemed to have a negative impact on patients with grade 2 and 3 gliomas (Figure 3A, HR = 3.26, 95%CI: 2.19–4.85).

#### The impact of non-SSRIs on overall survival of glioma patients.—

Non-SSRIs contained “nonselective monoamine reuptake inhibitors,” “Monoamine oxidase inhibitors, nonselective,” “Monoamine oxidase A inhibitors,” and “Other antidepressants” (ATC code: N06AA, N06AF, N06AG and N06AX). Three studies involving 2595 gliomas discussed about non-SSRIs. Figure 3B showed that non-SSRI use seemed to be unassociated with OS when taking these studies together (HR = 2.80, 95% CI: 0.86–9.09). Conclusions unfavorable to survival held when looking at GBM and LGG separately (Figure 3B, GBM: HR = 3.54, 95% CI: 2.51–4.99; LGG: HR = 7.71, 95% CI: 4.25–14.00). However, when limiting the drug class to tricyclics, the effect on OS became insignificant (HR = 0.83, 95% CI: 0.53–1.30).

#### The impact of antidepressants on GBM.—

Since there were 2 studies grouping all patients using antidepressants into a single category for survival analysis, and survival data for specific drug classes could not be obtained,^18,20^ in order to include data from these 2 studies, an analysis of the impact of antidepressants on GBM should be conducted, regardless of drug type. As in Edström S, et al.’s study, the same control group (GBM patients without antidepressant medication) was used for the analysis of the effects of SSRI and non-SSRI on GBM patients, these 2 sections could not be combined in one integrated analysis. Therefore, these 2 sections were merged with other studies, respectively, as represented in Figure 3C as subgroups 1 and 2. Results showed that when eliminating drug classification, antidepressants had no significant effect on GBM patients’ survival (Figure 3C, Subgroup 1: HR = 1.16, 95% CI: 0.69–1.93; Subgroup 2: HR = 1.18, 95% CI: 0.75–1.85), which highlighted the necessity of detailed antidepressants categorization.

## Discussion

In this systematic review and meta-analysis, we conducted an examination of 7 retrospective studies, comprising a total of 5579 patients. The conclusions drawn from these studies exhibited considerable heterogeneity. This variability may stem from the fact that, despite efforts to control for several common confounders, the included populations remained highly diverse, as will be discussed later. Following a comprehensive analysis, our findings suggested that within the context of GBM, the utilization of SSRIs appeared to yield no discernible impact on survival, whereas non-SSRIs exhibit adverse effects. Conversely, when considering LGG, both SSRIs and non-SSRIs usage demonstrated associations with poorer survival outcomes.

However, we believe all current retrospective studies suffer from baseline mismatch. Among the 7 studies included, the common inclusion strategy was to retrieve existing databases of glioma populations, then divide them based on antidepressant use and compare survival outcomes. Although baseline data like age and KPS were matched, a critical factor—depression status—was overlooked. Currently, it is a well-established conclusion that depression significantly worsens the survival of glioma patients.^21^ Therefore, antidepressant users, who often have more depressive symptoms, may inherently have worse survival outcomes, indicating baseline imbalance. This could explain why, despite the potential cytotoxic effects of antidepressants on tumors suggested by preclinical research, no improved survival was observed. Future cohort analyses should account for depression status in both treatment and control groups, possibly by matching patients based on depression scores measured using related depression scales.

Additionally, we suggest that future clinical studies should pay more attention to baseline details, such as the dosage of antidepressants and their concurrent use with TMZ therapy or radiotherapy. Unlike TMZ, which has a relatively fixed regimen, antidepressant treatment is highly individualized based on each patient’s depressive symptoms. This variability creates a highly heterogeneous cohort among glioma patients taking antidepressants, necessitating further subdivision for subgroup analysis—a consideration likely overlooked in any of the 7 included studies. Moreover, the cytotoxic effects of antidepressants on tumor cells can vary with concurrent therapies. For example, combination therapy with imipramine and TMZ has shown enhanced inhibitory effects on glioma growth compared to either agent alone, both in vitro and in vivo.^31^ Fluoxetine appears to sensitize glioma cells to TMZ through the CHOP-dependent apoptosis pathway^32^ and may enhance radiation-induced glioma cell death.^33^ Similarly, sertraline combined with TMZ produced a significant synergistic effect compared to individual treatments.^34^ Therefore, it is crucial to document whether antidepressants are used alone or in combination with other treatments and to compare survival outcomes accordingly.

Furthermore, clinical studies should explore the specific effects of different antidepressants. As demonstrated by Bi J, et al., fluoxetine use was associated with better prognosis in GBM patients, while citalopram and escitalopram, despite being SSRIs, did not significantly impact overall survival.^8^ This variability may reflect diverse interactions with cellular pathways in glioma cells; for example, fluoxetine inhibits oncogenic EGFR signaling more effectively than other SSRIs.^8^ This also explains why there was so much heterogeneity in the conclusions of the 7 included studies. Two of them grouped all antidepressants together,^18,20^ 3 distinguished between SSRIs and non-SSRIs,^5,16,17^ one focused on tricyclics,^19^ and only one was specific to a particular drug.^8^ This diversity in antidepressant classifications contributed to the heterogeneity of the findings. Focusing on specific drugs may also provide insights into molecular markers suitable for targeted therapies. For instance, fluoxetine might be particularly effective for gliomas with high EGFR pathway activation, as suggested by Junfeng Bi et al.‘s findings.

Despite multiple preclinical studies elucidating the functions of antidepressants in vitro and in vivo, there are significant differences between preclinical research and real-world drug application. Firstly, patients take antidepressants to alleviate depressive symptoms, but the actual dose that reaches the tumor microenvironment is unclear. The drug concentrations used in cellular experiments targeting glioma cells may differ significantly from those in the actual tumor microenvironment, necessitating consideration of the role of antidepressants in real physiological conditions. Secondly, the pharmacological actions of antidepressants, such as SSRIs increasing serotonin levels at synapses, must be considered. Given that elevated serotonin levels may stimulate cancer cell behavior,^35^ the impact of SSRIs on gliomas needs further exploration. Therefore, it is crucial to evaluate both the direct effects and pharmacological actions of antidepressants in tumor biology.

A biased assessment of the primary literature is critical for a meta-analysis. To evaluate the presence of publication bias, a funnel plot (Figure 2) was employed, and the results appeared to be satisfactory. However, several potential sources of bias warrant consideration. First is misclassification bias. All primary data were acquired through medical records. Having a prescription for antidepressants does not exactly indicate that the patient is actually taking antidepressants, which may lead to misclassification. Attrition bias is another concern, as differential dropout rates between study arms can affect results. Selection bias also needs attention. The study by Bi J, et al. using the IBM MarketScan insurance claims dataset only included patients under 65 years of age with commercial health insurance. Given that the median age of patients with GBM is around 65 years,^1^ this approach may exclude about 50% of eligible GBM patients. However, as shown in Table 2, the average age across the other 6 studies is approximately 60 years, which narrows the age gap between the patient cohort in Bi J, et al.‘s study and those in the other studies. This supports the inclusion of Bi et al.’s study in our systematic meta-analysis.

The potential impact of prevalent user bias is an important consideration. In all 7 studies, antidepressant use was defined by the presence of a prescription history following a glioma diagnosis. However, only the study by Edström S, et al. excluded patients with a history of antidepressant use prior to their glioma diagnosis; the other 6 studies did not exclude this group of patients. As no specific studies have explored the effects of prediagnosis antidepressant use on glioma outcomes as far as we know, we can only hypothesize about the potential impacts of this bias. One possibility is that the drug’s effect may change over time. Additionally, long-term antidepressant use before diagnosis could alter certain risk factors, potentially influencing prognosis. The exclusion of prevalent users may be related to the fact that Edström S, et al. reported a significantly higher HR among antidepressant users compared to other studies, suggesting that the findings of Edström S, et al. may more accurately reflect the true association, where the use of both SSRIs and non-SSRIs is linked to poorer outcomes.

## Conclusion

Following a systematic analysis of 7 observational, retrospective cohort studies, our findings suggested that for patients diagnosed with GBM, the utilization of antidepressant medications was not significantly associated with improved survival, and particularly, non-SSRI usage was correlated with poorer survival outcomes. In the case of LGG, the use of antidepressant medications seemed to correlate with poorer survival. Furthermore, our analysis emphasized the importance of distinguishing between different classes of antidepressants and their specific associations with various glioma subtypes. We provided guidelines for future research on how to address patient heterogeneity: conducting subgroup analyses by matching patients’ depressive states, recording antidepressant dosages along with concurrent treatment regimens, and distinguishing between different types of antidepressants, aiming to help further elucidate the true effects of antidepressants in the glioma population.

## Supplementary Material

vdae181_suppl_Supplementary_Table_S1

vdae181_suppl_Supplementary_Table_S2
