# Supplementary material for: Impact of antidepressant use on survival outcomes in glioma patients: A systematic review and meta-analysis
Source: Neurooncol Adv. 2024 Oct 26;6(1):vdae181. doi: 10.1093/noajnl/vdae181 (PMC11582889; doi:10.1093/noajnl/vdae181)
Supplement: vdae181_suppl_Supplementary_Table_S1 [file vdae181_suppl_supplementary_table_s1.docx]

**Supplementary Table 1. Strategies for assessing antidepressants use.**

| Study | Data Sources | Strategy |
| --- | --- | --- |
| Bi J, et al. 2021. | the IBM MarketScan insurance claims dataset | Use NDCs to query patient claims history for medications: fluoxetine (910 NDCs). |
| Caudill JS, et al. 2011. | Institutional records^a^ | By searching institutional records, patients who were taking an SSRI (ie, citalopram, escitalopram, fluoxetine, fluvoxamine, paroxetine, or sertraline) during the course of their treatment for GBM were identified. |
| Edström S, et al. 2023. | The RISK North database | The 1231 glioma cases were cross-referenced with the Prescribed drug register to identify prescriptions of antidepressant medications. Antidepressants were categorized into 2 groups: SSRI (ATC N06AB) and non-SSRI. In the non-SSRI group, N06A antidepressants other than N06AB SSRI were included (N06AA, selective monoamine reuptake inhibitors, N06AF monoamine oxidase inhibitors, nonselective, N06AG monoamine oxidase A inhibitors, and N06AX Other antidepressants). |
| Gramatzki D, et al. 2020. | The Cancer Registry of the Cantons Zurich and Zug | Data on use of antidepressant drugs were extracted from clinical records. No detailed search strategies are described. |
| Otto-Meyer S, et al. 2020. | The Northwestern Medicine Enterprise Data Warehouse (EDW) | SSRI use was searched for within the medication records of the EDW, and included both inpatient administration and reported outpatient prescriptions. SSRI search terms included the generic and primary brand name used in the United States and are as follows: (i) fluoxetine, Prozac; (ii) citalopram, Celexa; (iii) escitalopram, Lexapro; (iv) sertraline, Zoloft; (v) paroxetine, Paxil; (vi) vilazodone, Viibyrd. |
| Seliger C, et al. 2023. | The CENTRIC, CORE, AVAglio and ACT-IV trials | Data on use of antidepressant drugs were extracted from clinical records. Tricyclic antidepressants included doxepin, amitriptyline, imipramine, clomipramine, desipramine, nortriptyline, trimipramine and opipramol. Selective monoamine reuptake inhibitors included citalopram, escitalopram, sertraline, paroxetine, fluoxetine, fluvoxamine, venlafaxine, duloxetine, reboxetine and milnacipran. Other antidepressants included tranylcypromine, moclobemide, mianserin, mirtazapine, maprotiline, lithium, agomelatine, bupropion, trazodone or tianeptine. |
| Walker AJ, et al. 2012. | The General Practice Research Database | Data on use of antidepressant drugs were extracted from treatment records. The primary exposure was the use of tricyclic antidepressants [section 4.3.3 of the British National Formulary (BNF)]. To be exposed, a patient must have had a repeat prescription (≥2) within the period being examined for exposure. A period of 6 months post-diagnosis was used to determine drug exposure. |
