# Supplementary material for: Impact of antidepressant use on survival outcomes in glioma patients: A systematic review and meta-analysis
Source: Neurooncol Adv. 2024 Oct 26;6(1):vdae181. doi: 10.1093/noajnl/vdae181 (PMC11582889; doi:10.1093/noajnl/vdae181)
Supplement: vdae181_suppl_Supplementary_Table_S2 [file vdae181_suppl_supplementary_table_s2.docx]

**Supplementary Table 2. Adjusted Confounders.**

| Study | Adjusted Confounders |
| --- | --- |
| Bi J, et al. 2021. | A stricter propensity score-matched analysis of SSRI-treated and nontreated patients with GBM was performed, accounting for age, gender, and baseline comorbidities (each SSRI-treated patient is matched to two non-SSRI-treated patients). |
| Caudill JS, et al. 2011. | Age, RPA Classification, and Extent of Resection. |
| Edström S, et al. 2023. | Age, gender, and type of surgery. |
| Gramatzki D, et al. 2020. | Age, KPS, extent of resection, MGMT promoter methylation status, and postsurgical therapy. |
| Otto-Meyer S, et al. 2020. | Age, gender, and surgical intervention. |
| Seliger C, et al. 2023. | Age, gender, WHO performance status, steroid use, MGMT and extent of surgery. |
| Walker AJ, et al. 2012. | Age, gender, smoking, BMI, comorbidity (Charlson index), diagnosed depression, anxiety disorders, and insomnia. |
